# Smad2/3/4 complex could undergo liquid liquid phase separation and induce apoptosis through TAT in hepatocellular carcinoma

**DOI:** 10.1186/s12935-024-03353-x

**Published:** 2024-05-21

**Authors:** Jiong Li, Wendi Wang, Sang Li, Zhengkang Qiao, Haoyue Jiang, Xinyue Chang, Yaning Zhu, Hongpei Tan, Xiaoqian Ma, Yuqian Dong, Zhenhu He, Zhen Wang, Qin Liu, Shanhu Yao, Cejun Yang, Min Yang, Lu Cao, Juan Zhang, Wei Li, Wei Wang, Zhe Yang, Pengfei Rong

**Affiliations:** 1 Department of Radiology, The Third Xiangya Hospital, Central South University, Changsha, Hunan China; 2 Postdoctoral Station of Medical Aspects of Specific Environments, The Third Xiangya Hospital, Central South University, Changsha, Hunan China; 3 College of Life Science, Liaoning University, Shenyang, China; 4 Shenyang Key Laboratory of Chronic Disease Occurrence and Nutrition Intervention, College of Life Sciences, Liaoning University, Shenyang, 110036 China; 5 Engineering and Technology Research Center for Xenotransplantation of Human Province, Changsha, China

**Keywords:** Smad2/3/4 complex, HCC, LLPS, TAT, Caspase-9

## Abstract

**Background:**

Hepatocellular carcinoma (HCC) represents one of the most significant causes of mortality due to cancer-related deaths. It has been previously reported that the TGF-β signaling pathway may be associated with tumor progression. However, the relationship between TGF-β signaling pathway and HCC remains to be further elucidated. The objective of our research was to investigate the impact of TGF-β signaling pathway on HCC progression as well as the potential regulatory mechanism involved.

**Methods:**

We conducted a series of bioinformatics analyses to screen and filter the most relevant hub genes associated with HCC. *E. coli* was utilized to express recombinant protein, and the Ni–NTA column was employed for purification of the target protein. Liquid liquid phase separation (LLPS) of protein in vitro*,* and fluorescent recovery after photobleaching (FRAP) were utilized to verify whether the target proteins had the ability to drive force LLPS. Western blot and quantitative real-time polymerase chain reaction (qPCR) were utilized to assess gene expression levels. Transcription factor binding sites of DNA were identified by chromatin immunoprecipitation (CHIP) qPCR. Flow cytometry was employed to examine cell apoptosis. Knockdown of target genes was achieved through shRNA. Cell Counting Kit-8 (CCK-8), colony formation assays, and nude mice tumor transplantation were utilized to test cell proliferation ability in vitro and in vivo.

**Results:**

We found that Smad2/3/4 complex could regulate tyrosine aminotransferase (TAT) expression, and this regulation could relate to LLPS*.* CHIP qPCR results showed that the key targeted DNA binding site of Smad2/3/4 complex in *TAT* promoter region is −1032 to −1182. In addition. CCK-8, colony formation, and nude mice tumor transplantation assays showed that Smad2/3/4 complex could repress cell proliferation through TAT. Flow cytometry assay results showed that Smad2/3/4 complex could increase the apoptosis of hepatoma cells. Western blot results showed that Smad2/3/4 complex would active caspase-9 through TAT, which uncovered the mechanism of Smad2/3/4 complex inducing hepatoma cell apoptosis.

**Conclusion:**

This study proved that Smad2/3/4 complex could undergo LLPS to active TAT transcription, then active caspase-9 to induce hepatoma cell apoptosis in inhibiting HCC progress. The research further elucidate the relationship between TGF-β signaling pathway and HCC, which contributes to discover the mechanism of HCC development.

**Supplementary Information:**

The online version contains supplementary material available at 10.1186/s12935-024-03353-x.

## Background

Hepatocellular carcinoma (HCC) is the most prevalent liver malignancy and is among the top leading causes of cancer-related mortality [1–3]. It is reported that genetic mutations, chromosomal aberrations, molecular signaling pathways, and epigenetic deregulation have been implicated in HCC [4], but the mechanism of HCC development still remains ambiguity. Therefore, to reveal the mechanism of HCC development in a new sight is critical to solve this problem.

Previous studies showed that TGF-β signaling is a double edge sword in HCC development. As a tumor suppressor, it inhibits HCC development through upregulate the cyclin-dependent kinase (CDK) inhibitor genes p15, p21, and p27 [5]. As a tumor promotor, it induce HCC invasion and epithelial-to-mesenchymal transition (EMT) through active pro-EMT inducers, such as TWIST1, TCF3, E12, SNAI1, SLUG, ZEB1 [6–8]. These researches suggest that TGF-β signaling pathway may switch from being tumor-suppression to tumor-promoting in HCC progress [9].

It is reported that TGF-β signaling pathway is dysregulated in many cancers [10]. TGF-β can suppress tumors directly by regulating cell differentiation and apoptosis, or indirectly by suppressing inflammation and stroma-derived mitogens [11–14]. Surprisingly, the TGF-β signaling pathway can also induce tumor cell migration and stimulate the epithelial to mesenchymal transition [10, 11]. The TGF-β signaling pathway is activated by TGF-β binding to TGF-β type II and type I receptors (TβR II and TβR I), which are active serine/threonine kinases, then TβRI was phosphorylated. Activated TβRI phosphorylates Smad2 and Smad3, which assemble heterodimeric and trimeric complexes with Smad4. Smad2/3/4 complexes translocate to the nucleus and regulate target gene expression [8, 10].

Smad2/3 and Smad4 assemble Smad2/3/4 complex then translocate to the nucleus and regulate target gene expression when TGF-β signaling is activated [10]. Studies show that transcription factors can undergo LLPS with mediator to activate gene expression [15]. Transcription coactivators, such as BRD4 and MED1, can also form phase-separated droplets to control gene expression [16]. Smad2/3/4 complex is a well known transcription factor. In this study, we proved that Smad2/3/4 complex could undergo LLPS to active gene expression.

Phase separation takes part in some physiological activities [17–19]. Such as cytoskeleton formation [20], RNA metabolism [21], nucleolar formation [22] and transcription [15, 16]. LLPS can reflect the highly dynamic changes of macromolecular substances in solution. The phase separation of protein and protein (or protein and nucleic acid) can form liquid droplets to activate or inhibit biochemical reactions. Recently, LLPS is reported to have relationship with pathologic process, for instance, rare genetic disease [23], neurodegenerative disease [17] and cancer [24]. It is reported that LLPS can affect the progress of HCC. For example, LLPS of glycogen encapsulates YAP into glycogen droplets, inhibits the activation of Hippo pathway and drives the occurrence of HCC [24]. The Twist1-YY1-p300 complex promotes miR-9 expression through LLPS, stimulates hepatoma cell invasion and metastasis [25]. All these researches suggest that LLPS is related to HCC progress. In our research, we found that the LLPS of Smad2/3/4 complex could inhibit HCC progress.

Previous studies show that the disturbance of tyrosine metabolism is related to cancer progress [26]. In addition, clinical patients with hereditary tyrosinemia are more likely to develop HCC [27, 28]. In HCC patients, serum tyrosine is often upregulated [29, 30]. These suggest that tyrosine metabolism imbalance is related to HCC process. The *TAT* gene encodes a mitochondrial protein tyrosine aminotransferase which is present in liver. TAT involves in tyrosine breakdown and converts tyrosine to p-hydroxyphenylpyruvate. In previous research, TAT acts as a tumor suppressive player in HCC [31]. The regulatory mechanism of TAT expression still remains unknown. In our research, we found that Smad2/3/4 complex would undergo LLPS to regulate TAT expression.

In this study, we found that Smad2/3/4 may activate TAT expression through liquid liquid phase separation. The CCK-8 and colony formation assays results showed that Smad2/3/4 complex inhibited HCC progress was related to TAT. Western-Blot and Flow cytometry assays results suggested that Smad2/3/4 complex could active caspase-9 to improve hepatoma cell apoptosis through TAT. Our research further correlated TGF-β signaling pathway with apoptosis through TAT, enriched the function of LLPS, which brings new insights into the molecular mechanism of HCC.

## Materials and methods

### Western blot analysis

HUH7 and HepG2 cells were lysed with RIPA buffer. We used Future PAGE gel (ET12420gel; ACE Biotechnology, China) to separate protein. The eBlotTML1 machine (L00686C; Genescript, China) was used for transferred protein onto polyvinylidene fluoride membranes. The target proteins were detected using special antibody and visualized using the developer (UC279012; Thermo, USA). Antibody information: anti-β-actin antibody (AC038; Abclonal, Wuhan, China), anti- Smad2 antibody (A19114; Abclonal), anti-Smad3 antibody (A19115; Abclonala), anti-TAT antibody (A6764; Abclonal). anti-Smad4 antibody (A19116; Abclonala). anti-TAT antibody(A6764; Abclonal). anti-Caspase-9 antibody(A11910, A0281; Abclonala). Secondary antibodies: horseradish peroxidase (HRP)-conjugated goat anti-rabbit IgG (H + L) (AS014; Abclonal). Original data of Western blot were showed in supplementary material.

### QRT-PCR analysis

All cells and tumor tissues were lysed by RNAiso Plus (Takara Biotechnology, Dalian, China). Total RNA was extracted by chloroform and precipitated by isopropanol. The cDNA Synthesis Kit was used to synthesize first-strand cDNA (K1622, Thermo, USA). qRT-PCR was performed by PerfectStart® Green qPCR SuperMix according (AQ601, TransGen Biotech, Beijing, China) to the manufacturer’s protocol. The primers used were as follows: β-actin forward, 5’-GAGAAAATCTGGCACCACACC-3’, reverse, 5’-GGATAGCACAGCCTGGATAGCAA-3’; TAT forward, 5’- GAGTTCACGGAGCGGTTAGT-3’, reverse, 5’- ATCATCACCTCGGGGACTGT-3’; Smad2 forward, 5’-GCTTCTCTGAACAAACCAGGTC-3’, reverse, 5’-TGTGAAGATCAGGCCAGCG-3’; Smad3 forward, 5’-TCGTCCATCCTGCCTTTCAC-3’, reverse, 5’- CTGCCCCGTCTTCTTGAGTT-3’; Smad4 forward, 5’- ACTTTGAGGGACAGCCATCG-3’, reverse, 5’- GATGGGGCTAACAGAGCTGG;

### CCK-8 analysis

Transfect HUH7 and HepG2 cells with target vector via lip3000 (Followed the Reagent instructions). Inoculated cell suspension in 96-well plate (1000–5000 cells/well), Pre-incubate the plate in cell incubator at 37 ℃ according to experimental need. Add equal to 1/10 of the media volume CCK-8 (FC101-01, TransGen Biotech, Beijing, China) solution to each well and then incubate the plate in 37 ℃ for 1–3 h. Used a microplate reader to measure the absorbance at 450 nm.

### ELISA analysis

Treated cells were lysed using RIPA buffer, tumors were frozen with liquid nitrogen and grind, then also lysed using RIPA buffer. Lysed cell and tumor was centrifuged at high speed and collected supernatant. The supernatant was used Human TyR ELISA Kit (CB15676-Hu, Shanghai Coibo Bio Technology, Shanghai, China) to test the tyrosine content according to the manufacturer’s protocol. Using a microplate reader to measure the absorbance at 450 nm.

### Virus package

We used the lentiviral system to knockdown the target genes. Sh-vector (with special shRNA sequence) and other two plasmids pMD2.G and pSPAX2 were co-transfected into HEK293T cells by lip3000 (L3000075, Thermo, USA). The viral supernatant was collected at least 40 h after transfection. The lentivirus was used to infect HUH7 and HepG2 cells for at least 24 h, and screened at least 7d with puromycin after infection. Western bolt and qPCR were used to check the knockdown efficiency. The shRNA sequence used for target gene konockdown were as follows: shRNA-Smad2-1: 5’-CAAGTACTCCTTGCTGGATTG-3’; shRNA-Smad2-2: 5’-GCGTTGCTCAAGCATGTCATA-3’; shRNA-Smad3-1: 5’-GAGCCTGGTCAAGAAACTCAA-3’; shRNA-Smad3-2: 5’-GCCTCAGTGACAGCGCTATTT-3’; shRNA-Smad4-1: 5’- GTACTTCATACCATGCCGATT-3’; shRNA-Smad4-2: 5’- GCTGCTGGAATTGGTGTTGAT-3’.

### Recombinant protein expression and purify

We used *E. coli* to express recombinant protein (Tagged with EGFP/mCherry/EBFP), and protein (His tagged) was purified with Ni–NTA column. Target vector was transfected into *E. coli* and cultured in LB medium. Add IPTG to final concentration 0.24 mg/ml when medium OD value to 0.6–0.8. Continuing cultivate the *E. coli* for 16–24 h in 16–24 ℃, then collect the *E. coli.* Treat the bacteria with ultrasonic crusher (200W, 3 s on and 4 s off). Follow the protocol of Ni–NTA column to purify protein.

### Phase separation in vitro

Purified proteins were diluted in PBS, added PEG8000 at a final concentration 10% (M/V). The solution is expected to turn cloudy if the peptide is prone to liquid liquid phase separation. Use different NaCl concentration and temperature to test the environmental effect of liquid droplets formation. The liquid droplets were used detected by fluorescence microscope or laser confocal microscope.

### Flow cytometry

Transfect HUH7 and HepG2 cells with vectors through lip3000, and knock down target genes by lentivirus (shRNA). To test hepatoma cell apoptosis, a total of 4 × 10^5^ cells per sample were prepared as signal-cell suspensions of the treated cells, and stained using the Annexin V-FITC Apoptosis Detection Kit(Cat: 556547, BD Bioscience, USA). The cells were analyzed by flow cytometry. Raw data were analyzed through the FlowJo_V10.8.1 software.

### Tumor formation in nude mice

HUH7 cells well cultured in DMEM medium with 10% FBS and 1% penicillin streptomycin. Knock down of target genes by shRNA and screened at least 7d with puromycin. Pancreatic enzyme digestion the cells and count the numbers. Resuspend cells with the sodium chloride (0.9%). 1.5–2 × 106 tredted cells were injected in one 6-8w nude mice. Start record the tumor growth when the tumor length grown to 3–5 mm. When tumor volume growth to 1000–1500 mm^3^, all mice were sacrificed and the tumor tissues were used for further study.

### CHIP qPCR

Collected hepatoma cells (HUH7 and HepG2), about 1–2 × 10^7^ per sample. Wash the cells with PBS once, and fix cells by adding pre-warmed plain media supplement with the following reagents: Formaldehyde(1%), EDTA (pH 8.0, 0.15 mM), EGTA (pH 8.0, 0.075 mM), Hepes(pH 8.0, 0.015 mM). Keep cells at room temperature for 10 min to allow cross-linking, add glycine to a final concentration of 0.125 M to stop cross-linking. Rinse cells with cold PBS. Resuspend cells with BufferI(Hepes–KOH, PH7.5, 50 mM; NaCl, 140 mM; EDTA, 1 mM; glycerol, 10%; NP-40, 0.5%; Triton X-100, 0.25%). Rock at 4 ℃ for 10 min and spinning at 3000 rpm, 5 min. Resuspend cells with BufferII(NaCl, 200 mM; EDTA pH 8.0, 1 mM; EGTA pH 8.0, 0.5 mM; Tris pH 8.0, 10 mM). Rock at 4 ℃ for 10 min and spinning at 3000 rpm, 5 min. Resuspend cells with BufferIII(EDTA pH 8.0, 1 mM; EGTA pH 8.0, 0.5 mM; Tris pH 8.0, 10 mM; N-lauroyl-sarcosine, 0.5%). Sonicate the samples (30 s on 30 s off). Run 1% agarose gel with 10 ul of chromatin. A smear should be seen, that spans from 200 bp to 1 kb. Spin 14000 rpm, 10 min in microfuge at 4 ℃. TE buffer (Tris–HCl pH8.0, 10 mM; EDTA pH8.0, 1 mM) wash the ProteinA/G beads and BSA(1%) blocked it. Added the target antibody and rock in 4 ℃ for 16-24 h. Wash the beads with RIPA(Hepes pH 7.6, 50 mM; EDTA,10 mM; DOC, 0.7%; NP-40, 1%; LiCl, 0.5 mM) for 3 times. Wash the beads with Buffer (10 mM Tris pH 8.0/1 mM EDTA/ 50 mM NaCl) for 3 times. Resuspend sample with Buffer (50 mM Tris pH 8.0, 10 mM EDTA, 1% SDS). Incubate at 65℃ for 15 min., vortex shock every 4 min. Spin to remove all beads and incubate the supernatant at 65℃ for 16–24 h. Add RNase A to the sample and incubate at 37 oC for 2 h. Add Proteinase K and incubate at 55℃ for 30 min. Gel recycling kit for recovering DNA fragments. The final samples were detected by qPCR. CHIP qPCR primer sequence were as follows. Site1: forward, 5’-TCTATTTGAATTTATTATAT-3’, reverse, 5’-CAAGGGCATCCTTGTCATAA-3’; Site2: forward, 5’-GCCCAAGACGTGAATAATTT-3’, reverse, 5’-CTCCAAGACCTCCAGTGGAT; Site3: forward, 5’-TGTGCTCCCTGTGGATAAGG-3’, reverse, 5’-AGTGATCTCCCCAGGGCTCA; Site4: forward, 5’-GAGGCTTCTCTTAACCCTTC-3’, reverse, 5’-ACCTCCTATGGTTGTTGGAA.

### Cell culture

HUH7 and HepG2 cell lines were cultured in DMEM with 10% FBS and 1% Penicillin streptomycin. Environment with 5% CO_2_ and hold on 37 ℃. Unless special note, most experiments of Smad2/3/4 complex activation were needed to add TGF-β1, the TGF-β1 concentration was 100-200PM.

## Results

### TAT expression is related with Smad2 and Smad3 in HCC

**Fig. 1:**
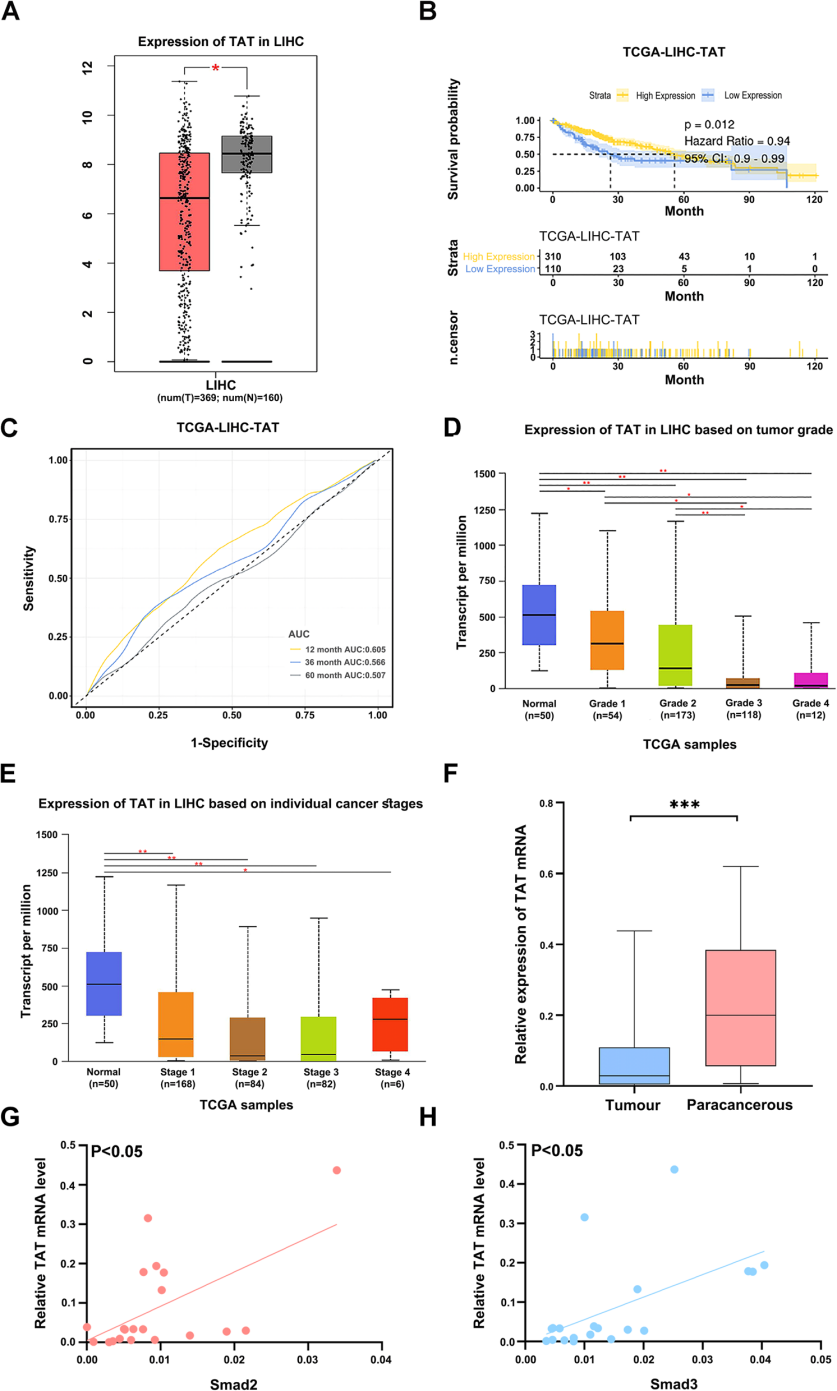
The mRNA level of TAT is positiv with Smad2 and Smad3. **A** Verification of the mRNA expression levels of TAT. Red represents tumor tissue, black represents normal tissue. **B** RFS and univariate COX analysis of TAT. **C** The ROC curve of TAT in HCC. **D** Expression of TAT in LIHC based on tumor grade. **E** Expression of TAT in LIHC based on individual cancer stages. **F** Expression level of TAT in HCC tissues and paracancerous n = 28. **G**–**H** The mRNA expression level relationship of TAT and Smad2, Smad3 in HCC tissues, n = 20. *P* < 0.05, *; *P* < 0.01,**; *P* < 0.001, ***

In our research, we used the GEPIA database to analyze the expression levels of TAT in HCC patients. The results showed that TAT was differentially expressed in these two groups (Fig. 1A). Subsequently, a univariate COX prognostic analysis was performed, and TAT gene was identified as having a significant impact on survival prognosis in HCC, indicating that high expression of the TAT gene is associated with better survival prognosis (Fig. 1B). In addition, ROC curves were generated for 1, 3, and 5 years (Fig. 1C). Our data also showed that low mRNA expression of TAT in HCC samples was significantly associated with mild clinical stage and pathological grade (Fig. 1D, E). Furthermore, the mRNA levels of TAT were down-regulated in liver cancer tissues compared with paracancerous (Fig. 1F). These results suggest that TAT may play an inhibitory role in HCC progression. Previous research also has reported that mutation of *TAT* can advance HCC progression [31]. However, the regulatory mechanism of TAT in HCC is unknown. In our research, we observed a positive correlation between TAT mRNA expression and Smad2 and Smad3 in clinical HCC tissues (Fig. 1G, H). Taken together, our results indicate a tumor suppressive role of TAT in HCC progression, and a regulatory role of the TGF-β pathway in TAT expression.

### TAT expression is regulated by Smad2/3/4 complex in HCC

**Fig. 2:**
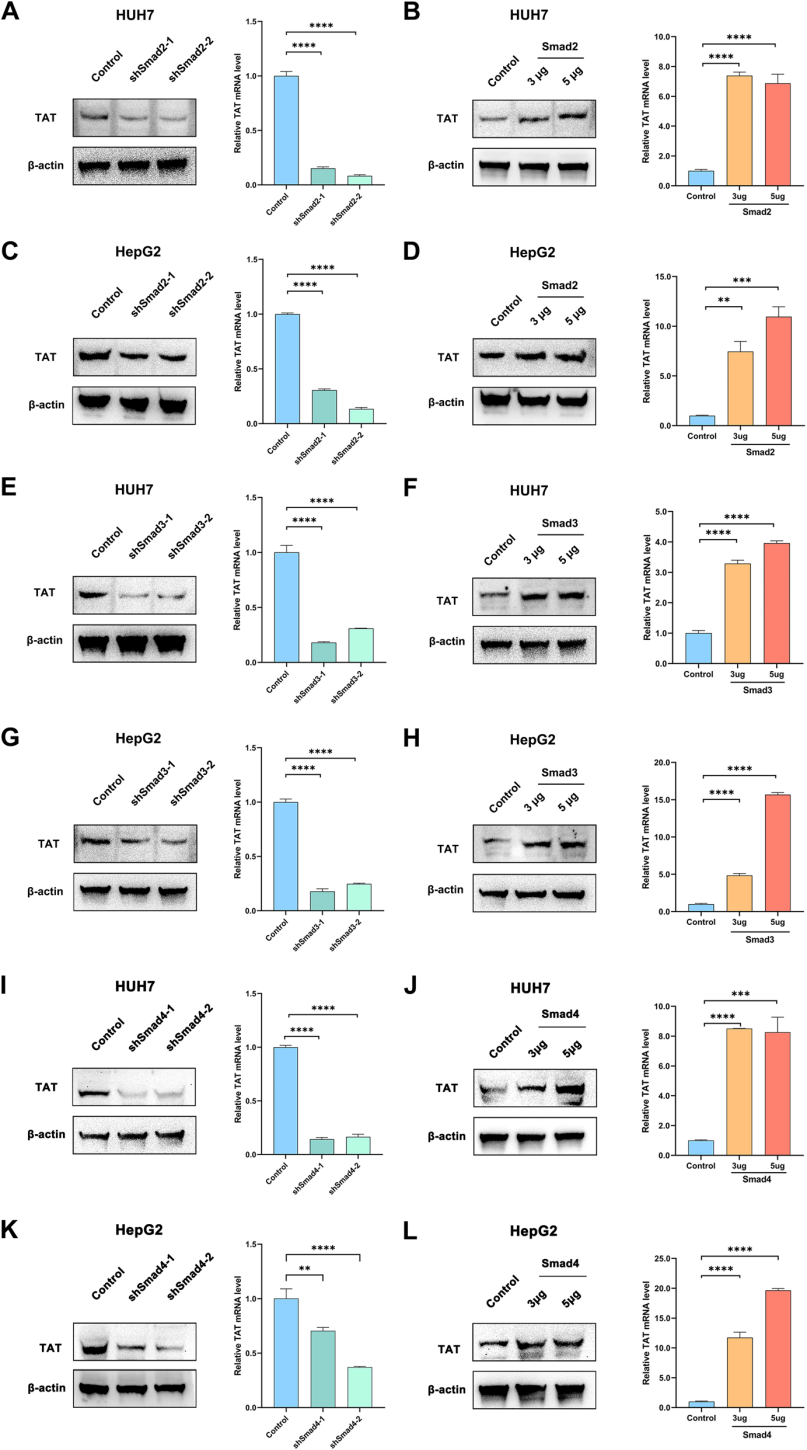
Smad2/3 complex upregulation TAT in HCC cell lines. **A**–**B** Relative mRNA and protein levels of TAT were detected by qPCR and western blot analysis in HUH7 which knockdown (**A**) or overexpress (**B**) Smad2. **C**–**D** Relative mRNA and protein levels of TAT were detected by qPCR and western blot analysis in HepG2 cells which knockdown (**C**) or overexpress (**D**) Smad2. **E**–**F** Relative mRNA and protein levels of TAT were detected by qPCR and western blot analysis in and HUH7 cells which knockdown (**E**) or overexpress (**F**) Smad3. **G**–**H** Relative mRNA and protein levels of TAT were detected by qPCR and western blot analysis in HepG2 cells which knockdown (**G**) or overexpress (**H**) Smad3. **I**–**J** Relative mRNA and protein levels of TAT were detected by qPCR and western blot analysis in HUH7 cells which knockdown (**I**) or overexpress (**J**) Smad4. **K**–**L** Relative mRNA and protein levels of TAT were detected by qPCR and western blot analysis in HepG2 cells which knockdown (**K**) or overexpress (**L**) Smad4. 3μg and 5 μg is represented the quality of overexpression vector in each hole of 6-Wells plats. TGF-β1 (150 pM) was added in medium to activate Smads. *P* < 0.05, *; *P* < 0.01,**; *P* < 0.001,***; *P* < 0.0001, ****

When TGF-β signaling is activated, Smad2/3 and Smad4 assemble into the Smad2/3/4 complex, which then translocates to the nucleus to regulate target gene expression [10]. In the aforementioned study, we observed a positive correlation between TAT expression and Smad2 and Smad3. Based on these findings, we postulated that the Smad2/3/4 complex might play a regulatory role in TAT expression in HCC cell lines. To further investigate this relationship, we successfully downregulated Smad2 using shRNA in HUH7 and HepG2 cell lines. Additionally, we overexpressed Smad2 in these cell lines (Supplementary Fig. S1A-D). QPCR and Western Blot analysis showed that knockdown of Smad2 downregulated the mRNA and protein expression of TAT in HUH7 and HepG2 cell lines (Fig. 2A–D). Further, we depressed and overexpressed Smad3 and Smad4, respectively (Supplementary Fig. S1E-L). The results showed that, the same as Smad2, Smad3 and Smad4 could also regulate TAT expression at both mRNA and protein level in HUH7 and HepG2 cell lines (Fig. 2E–L). These results suggest that TAT expression is regulated by Smad2/3/4 complex in hepatoma cells.

### Smad2/3/4 complex undergoes liquid liquid phase separation

**Fig. 3:**
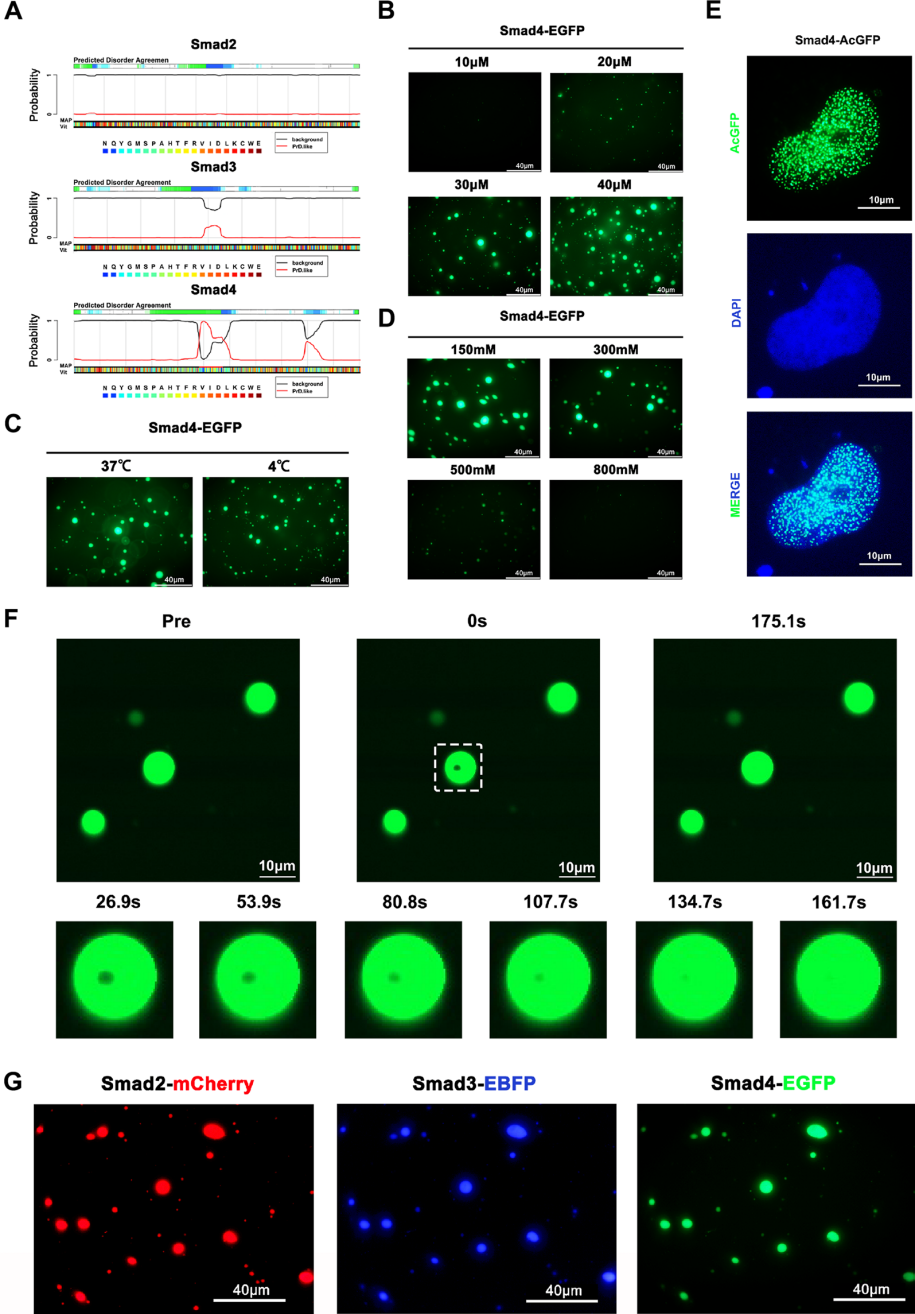
Liquid liquid phase separation of Smad2/3/4 complex. **A** Distribution of disorder and PrLD domain in Smad2, Smad3, Smad4. **B** Phase separation of Smad4 in vitro at different concentrations (from 10 to 40 µM in PBS). **C** Phase separation of Smad4 (30 µM) in vitro at different temperatures (37 ℃ and 4 ℃, 1 × PBS). **D** Phase separation of Smad4 (30 µM) in vitro at different NaCl concentrations (NaCl concentration 150, 300, 500, and 800 mM). **E** Smad4 undergo phase separation in HUH7 cells, TGF-β1 (10 ng/ml) treated cells for at least 12 h. TGF-β1 (200 pM) was added in medium to activate Smads. **F** Verified the liquid character of Smad4 droplets by Fluorescence recovery after photobleaching (FRAP). **G** Mixed Smad2, Smad3, Smad4 solution undergo LLPS in vitro

Smad2/3 and Smad4 assemble into a complex and translocate to the nucleus to regulate target gene expression when TGF-β signaling is activated [10]. Previous studies have shown that transcription factors undergo phase separation to regulate gene expression, and proteins that are prone to this process contain specific domains [15]. In particular, disorder sequences and PrLD domains are important for phase separation [17]. We analyzed the sequence characteristics of Smad2, Smad3, and Smad4, and found that Smad2 and Smad3 had little disorder sequence and no PrLD domain, while Smad4 had a large amount of disorder sequence and a PrLD domain (Fig. 3A). These findings suggest that the Smad2/3/4 complex may undergo LLPS during gene regulation, and that Smad4 may drive this process.

Further, we purified Smad4 (Tagged with EGFP) protein using *E. coli*. We observed that Smad4 formed droplets in vitro (Fig. 3B). The formation of these droplets was affected by temperature, indicating that this was an example of a lower critical solution temperature phase diagram (Fig. 3C). NaCl concentration also influenced the formation of the droplets, suggesting that LLPS was driven by electrostatic interactions between the PrLD and disorder sequence (Fig. 3D). In vivo, we detected the formation of Smad4 liquid droplets form in HUH7 cells (Fig. 3E). To verify the liquid nature of these droplets, we conducted an experiment using fluorescent recovery after photobleaching (FRAP). The FRAP results revealed that the droplets exhibited rapid fluorescence recovery following bleaching (Fig. 3F), indicating that these droplets possessed liquid characteristics and indicating that Smad4 underwent liquid–liquid phase separation (LLPS). Additionally, we purified Smad2 and Smad3 using *E. coli* and found that the mixed Smad2/3/4 complex underwent phase separation to form liquid droplets in vitro (Fig. 3G). Collectively, these findings indicate that the Smad2/3/4 complex undergoes liquid–liquid phase separation in Hepatoma cells. Previous studies have established that the Smad2/3/4 complex regulates target gene expression [10], and that LLPS of transcription factors is necessary for gene expression [15]. Our research has found that the Smad2/3/4 complex undergoes phase separation, we believe that it also undergoes phase separation during transcription activity.

### Smad2/3/4 complex binds to the TAT gene promotor site

**Fig. 4:**
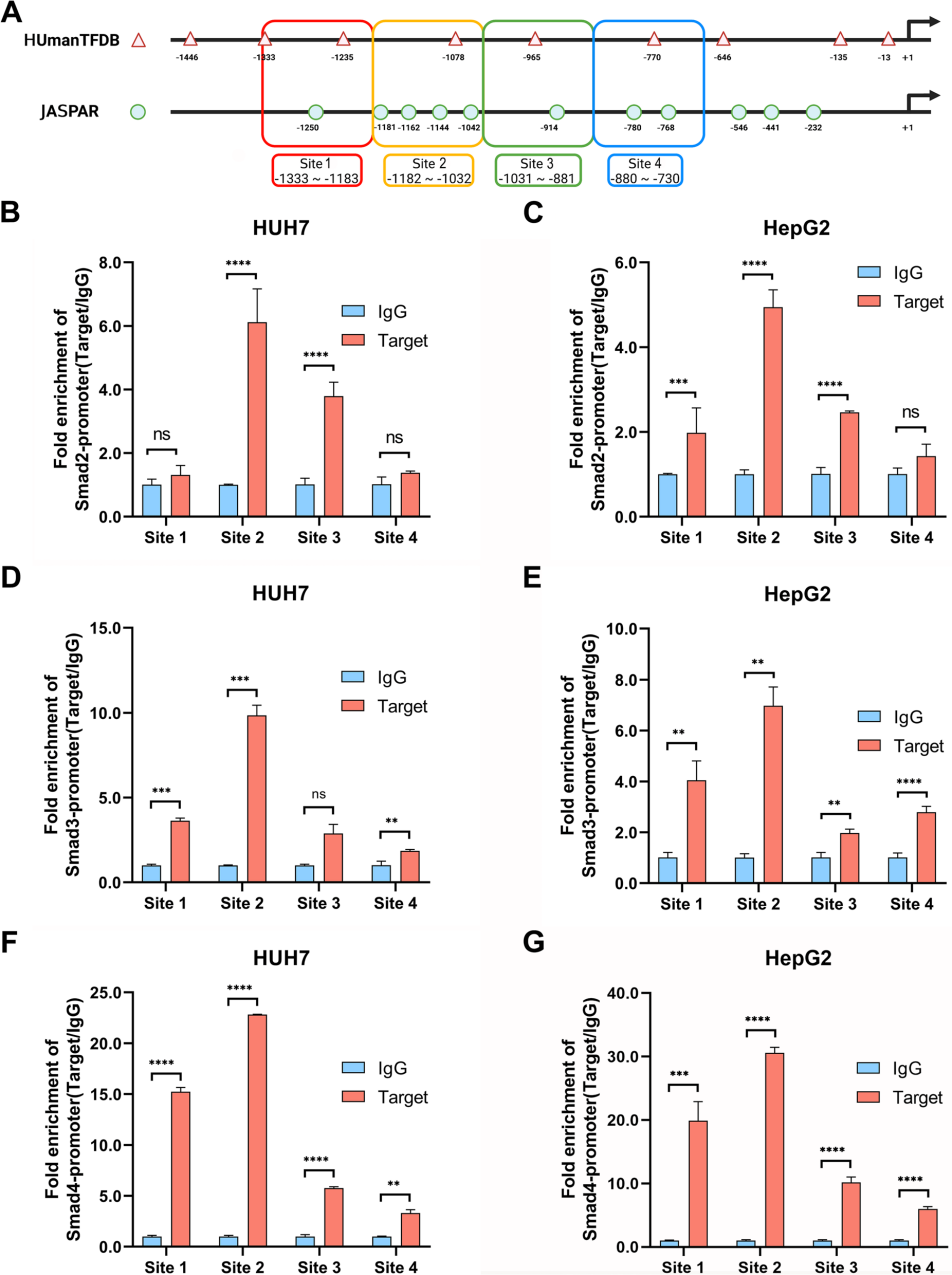
CHIP qPCR showed that Smad2/3 complex could bind to transcriptional regulatory regions of *TAT*. **A** Predicted the binding site of Smad2/3/4 complex in *TAT* transcriptional regulatory region. **B**–**C** CHIP qPCR analysis the binding sites of Smad2 in HUH7 (**B**) and HepG2 (**C**) cells. **D**–**E** CHIP qPCR analysis the binding sites of Smad3 in HUH7 (**D**) and HepG2 (**E**) cells. **F**–**G** CHIP qPCR analysis the binding sites of Smad4 in HUH7 (**F**) and HepG2 (**G**) cells. TGF-β1 (200 pM) was added in medium to activate Smads. *P* < 0.05, *;* P* < 0.01,**; *P* < 0.001,***; *P* < 0.0001, ****

Previous studies showed that Smad2/3/4 complex could bind to target gene promoter and increase promoter activity [32]. In our research, we used bioinformatics analysis found that Smad2/3/4 complex might bind to the transcriptional sites of TAT (Fig. 4A), suggesting that Smad2/3/4 complex can regulate the expression of TAT.

We used antibodies against Smad2, Smad3, and Smad4 to conduct chromatin immunoprecipitation (CHIP) experiments and qPCR to identify potential binding sites. The results indicated that the location between -1182 to -1032 is the key binding site of Smad2/3/4 complex in HUH7 and HepG2 cell lines (Fig. 4B–G). Above all, these data shows that Smad2/3/4 complex can bind to the *TAT* gene promotor site and undergo LLPS to regulate *TAT* gene expression.

### Smad2/3/4 complex regulate HCC progression through TAT

**Fig. 5:**
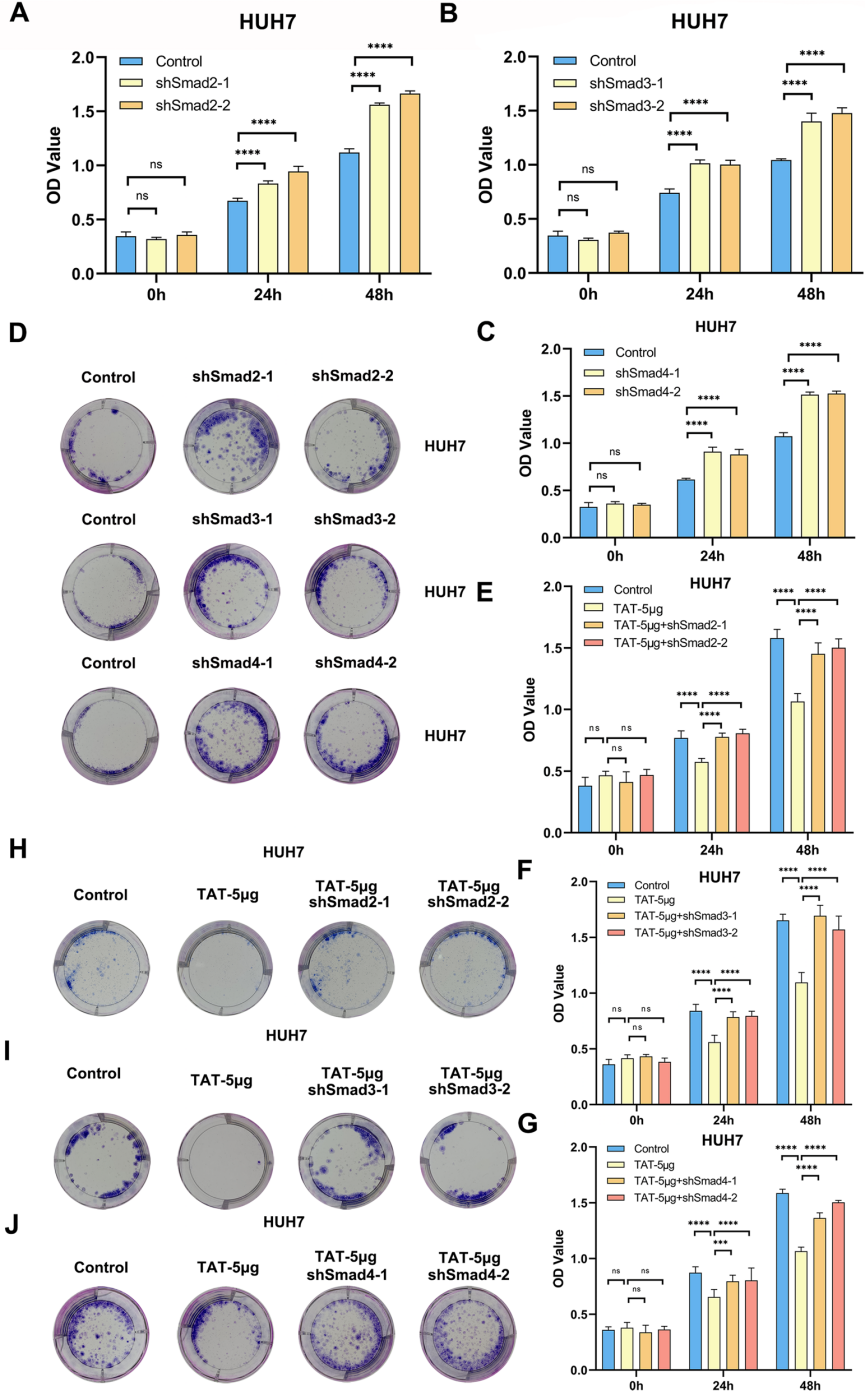
Smad2/3/4 complex could regulate HCC cell proliferation through TAT. **A**–**C** CCK analysis showed that knock down Smad2 (**A**), Smad3 (**B**) and Smad4 (**C**) could promote HUH7 cells proliferation. **D** Colony formation analysis showed that knock down Smad2, Smad3 and Smad4 could promote HUH7 cells proliferation. **E**–**G** CCK analysis showed that overexpressing TAT could inhibit HUH7 cells proliferation and knock down Smad2 (**E**), Smad3 (**F**) and Smad4 (**G**) could induce a compensatory effect. **H**–**J** Colony formation analysis showed that overexpressing TAT could inhibit HUH7 cells proliferation and knock down Smad2 (**H**), Smad3 (**I**) and Smad4 (**J**) could induce a compensatory effect. 5μg is represented the quality of overexpression vector in each hole of 6-Wells plats. TGF-β1 (100 pM) was added in medium to activate Smads. *P *< 0.05, *; *P* < 0.01,**; *P* < 0.001,***; *P* < 0.0001, ****

Previous study shows that mutation of TAT could promote HCC progression [31]. TGF-β signaling pathway could also affect tumor progress [10]. Our results showed that Smad2/3/4 could regulate the expression of TAT. We deduced that Smad2/3/4 could regulate HCC progress through TAT. To explore the relationship between TAT and Smad2/3/4 complex in HCC progression. We used CCK and clone formation assays to test the cell proliferation ability. The CCK assay results showed that Smad2/3/4 complex could inhibit hepatoma cell proliferation (Fig. 5A–C, Supplementary Fig. S2A-C). So as the clone formation assay results (Fig. 5D, Supplementary Fig. S2D). Overexpression of TAT could also inhibit HUH7 and HepG2 cell proliferation, downregulation of Smad2 could rescue the phenomenon, furthermore, the results of Smad3 and Smad4 were similar with Smad2 (Fig. 5E–G, Supplementary Fig. S2E-G). The tendency of clone formation assay results was the same as CCK assay results (Fig. 5H–J, Supplementary Fig. S2H-J).

**Fig. 6:**
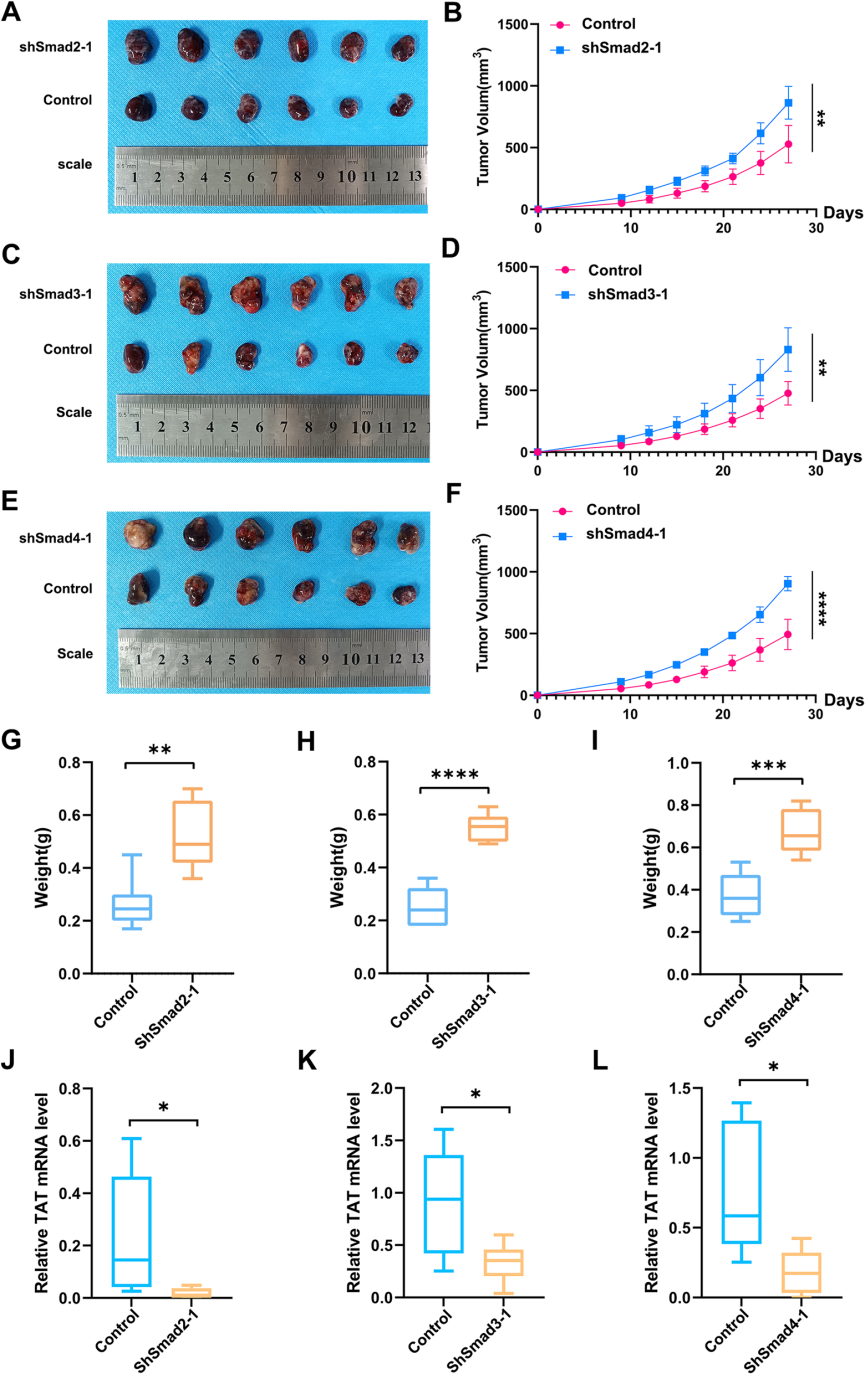
Knocking down Smad2, Smad3 and Smad4 could promote HCC progression. **A** Tumors from the nude mice to show the Smad2 effect on tumor progression. **B** Tumor growth curves of (**A**). **C** Tumors from the nude mice to show the Smad3 effect on tumor progression. **D** Tumor growth curves of (**C**). **E** Tumors from the nude mice to show the Smad4 effect on tumor progression. **F** Tumor growth curves of (**D**). **G**–**I** Tumor weight of each group, shSmad2 (**G**), shSmad3 (**H**), shSmad4 (**I**). **J**–**L** Tested TAT mRNA level of tumors from nude mice, shSmad2 (**J**), shSmad3 (**K**), shSmad4 (**L**). *P* < 0.05, *; *P* < 0.01,**; *P* < 0.001,***; *P* < 0.0001, ****

In nude mice tumor formation model, the tumor grew faster when breaking the Smad2/3/4 complex formation. We knocked down Smad2, Smad3 and Smad4, respectively. The tumor volume was obviously increased (Fig. 6A–F), so as the tumor weight (Fig. 6G–I). We use tumor tissues taken form the nude mice tumors to test the TAT expression level and tyrosine level. The results showed that knockdown Smad2, Smad3 or Smad4 could decrease the expression level of TAT (Fig. 6J–L). These results suggest that Smad2/3/4 complex regulates tumor formation directly through regulating TAT.

### Smad2/3/4 complex induces hepatoma cell apoptosis through regulating TAT

**Fig. 7:**
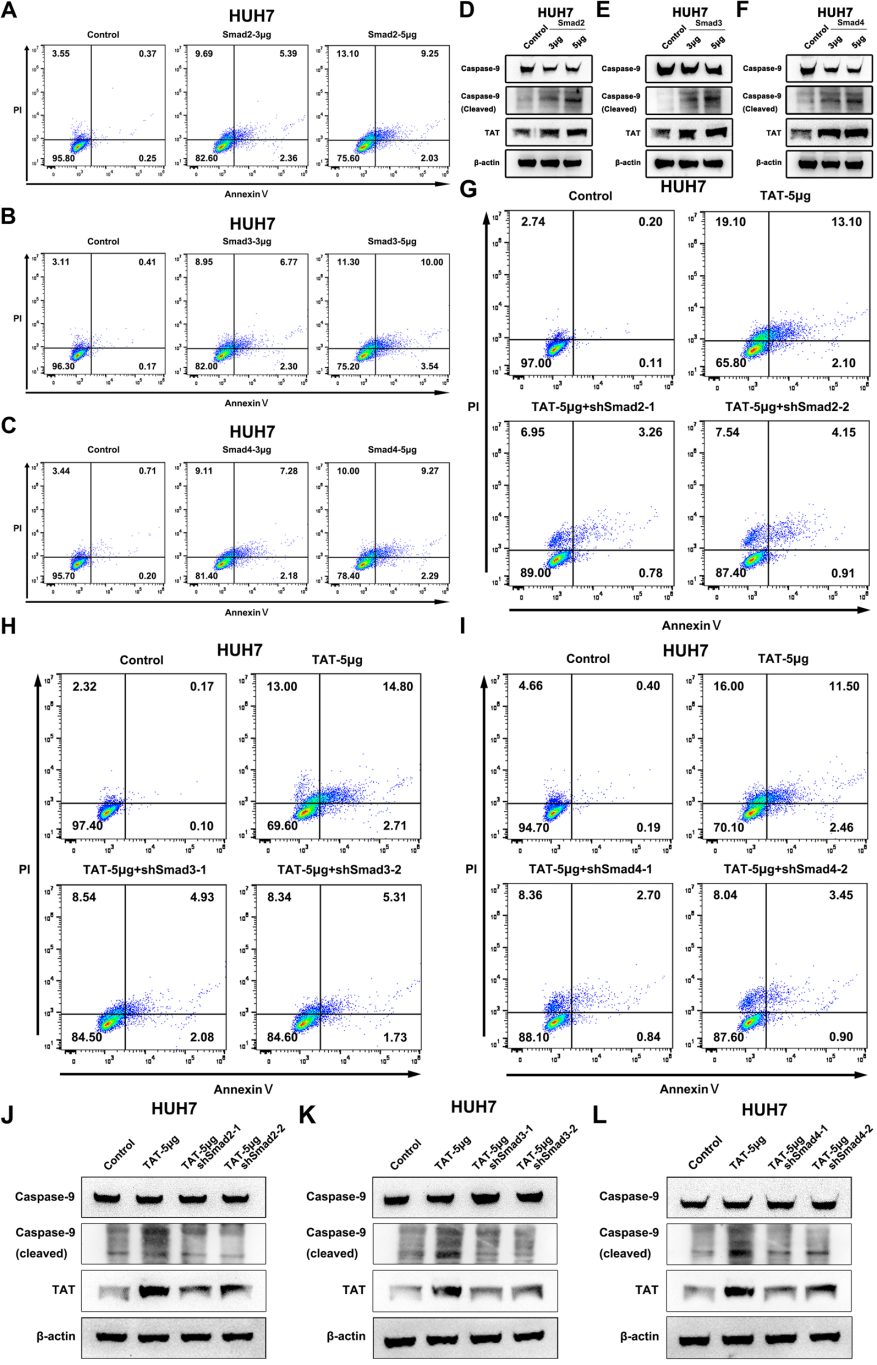
Smad2/3/4 could active caspase-9 through TAT in HUH7 cells. **A**–**C** Flow cytometry analysis showed that upregulated Smad2 (**A**), Smad3 (**B**) and Smad4 (**C**) could induce HUH7 cell apoptosis. **D**–**F** Upregulated Smad2 (**D**), Smad3 (**E**) and Smad4 (**F**) could active caspase-9 in HUH7 cells. **G**–**I** Flow cytometry analysis showed that upregulated TAT could induce apoptosis, but knocking down Smad2 (**G**), Smad3 (**H**), Smad4 (**I**) could inhibit apoptosis in HUH7 cells. **J**–**L** Upregulated TAT could active caspase-9, but knocking down Smad2 (**J**), Smad3 (**K**), Smad4 (**L**) could inhibit the caspase-9 active in HUH7 cells. 3μg and 5μg is represented the quality of overexpression vector in each hole of 6-Wells plats. TGF-β1 (100 pM) was added in medium to activate Smads

Previous study shows that TGF-β signaling pathway can regulate cell apoptosis by upregulating BIM, BMF, DAPK expression [10], and TAT induces hepatoma cell apoptosis by activating caspase-9 [31]. In our research, we found that Smad2/3/4 complex could undergo LLPS to regulate TAT expression. Probably, Smad2/3/4 complex could active caspase-9 through TAT to induce hepatoma cell apoptosis. We overexpressed Smad2, Smad3 and Smad4, respectively. Flow cytometry analysis showed that Smad2/3/4 complex could increase apoptosis of hepatoma cells (Fig. 7A–C, Supplementary Fig. S3A-C). Western blot results showed that, all three members could increase the expression of cleaved caspase-9 in hepatoma cell lines (Fig. 7D–F, Supplementary Fig. S3D-F). Further, flow cytometry analysis results showed that overexpression of TAT could increase apoptosis of hepatoma cells and knocking down Smad2 could create a compensatory effect to decrease hepatoma cell apoptosis. Similar phenomena were observed in Smad3 and Smad4 (Fig. 6G–I, Supplementary Fig. S3G-I). We discovered that overexpressed TAT could increase the expression of cleaved caspase-9, but knocking down Smad2 could decrease cleaved caspase-9 level in hepatoma cell lines, so as Smad3 and Smad4 (Fig. 7J–L, Supplementary Fig. S3J-L). All these results suggest that Smad2/3/4 complex could active caspase-9 and induce hepatoma cell apoptosis through regulating TAT.

**Fig. 8:**
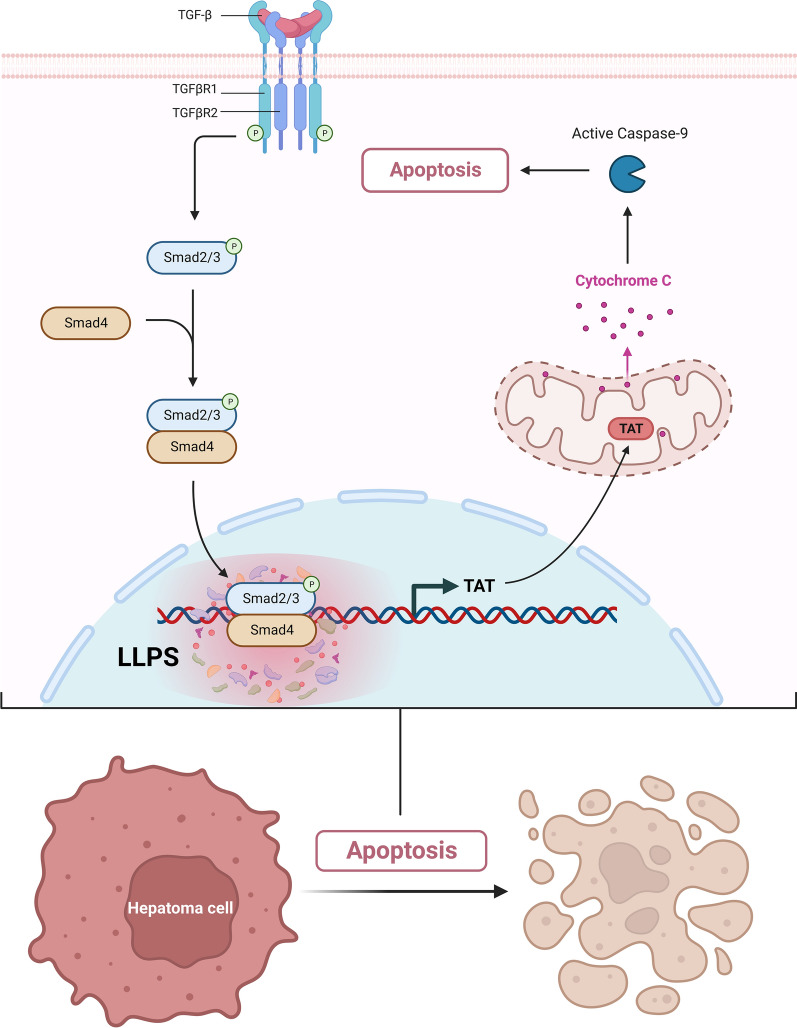
Working Model. The TGF-β signaling pathway is activated by TGF-β binding to TβRII, which recruits and phosphorylates TβRI, and then TβRI phosphorylates Smad2 and Smad3, which assemble heterodimeric and trimeric complexes with Smad4. Smad2/3/4 complexes translocate to the nucleus. In the nucleus, Smad2/3/4 complex could undergo LLPS to activate *TAT* transcription, then active caspase-9 to induce hepatoma cell apoptosis to inhibit HCC progression. The graph was created with BioRender.com

Taking together, our research explored that Smad2/3/4 complex could undergo LLPS and binds to TAT gene promotor site to upregulate TAT expression, which can active caspase-9 to induce hepatoma cell apoptosis (Fig. 8).

## Discussion

Numerous studies have shown that the accumulation of cellular and molecular aberrations can lead to the development of HCC, including abnormalities in epigenetics, transcriptome, proteome and metabolome [33]. Obviously, HCC has remarkable molecular heterogeneity including many genetic and protein-level changes. Therefore, the search for new targeted genes in HCC patients are expected to provide new directions to explore the mechanisms of HCC, as well as new drug targets.

TGF-β signaling pathway plays a dual role in cancer progress, not only inhibits tumor progress by inducing cell cycle arrest, but also induces tumor migration and stimulates epithelial to mesenchymal transition [10]. Activation of TGF signaling pathway dependent on receptor TGFβ receptors, but TGFβ receptors (TβR II and TβR I) behave very differently in HCC and other signaling pathway. TβR II mutation occurs in many cancers, such as colon, gastric, pulmonary, ovarian, esophageal, carcinomas [34]. TβR I and TβR II often down-regulated in lung, gastric, prostate, and bladder cancers, and TβR I promoter often methylated in gastric cancer [11]. TGFβ receptors also play significant roles in stromal cells in the tumor microenvironment. TGF-β showed immunosuppressive effects on both innate and adaptive immune cells [10]. The down expression and mutated of TβRII also fund in HCC [7, 8], but TβR II and TβR I also related with fibrogenesis and carcinogenesis (fibro-carcinogenesis) in HCC. During hepatitis virus–related chronic liver diseases, alterations additively shift hepatocytic Smad phospho-isoform signaling from tumor suppression to fibro-carcinogenesis, thereby accelerating liver fibrosis and increasing risk of HCC [35]. So, TGF-β signaling pathway showed complexity function in tumor progression. We supposed that Smad2/3/4 could regulate different gene expressions then regulate HCC progress. In our research, Smad2/3/4 complex played a suppression role in HCC through regulating TAT expression.

LLPS is a newly found phenomenon with a variety of possible functions, such as transcriptional or translational regulation [36–38], RNA metabolism [39], and signal transduction [40]. LLPS can locally concentrate molecules in condensates to activate reactions and signaling processes, increasing the local concentration of key enzymes or protein complexes to accelerate biochemical reactions [17]. Interestingly, we analysed the amino acid sequence characters of Smad2, Smad3 and Smad4. The data showed that Smad4 had large amounts of disorder sequence and PrLD domain, which could drive protein to undergo LLPS [36, 41, 42], suggesting that Smad4 could undergo phase separation. Further, we discovered Smad4 could form liquid droplets at different concentration, and the formation of liquid droplets could be affected by temperature and NaCl concentration. The mixed Smad2, Smad3 and Smad4 solution could also form liquid droplets. All these results showed that Smad2/3/4 complex could undergo phase separation. Previous studies show that Smad2, Smad3 and Smad4 are phosphorylated when TGF-β signaling pathway is activated [10]. So, Smad2/3/4 complex must stay on phosphorylation status when the complex translocates into the nucleus and regulates target gene expression. Phosphorylation and methylation are important for protein undergoing LLPS [43–46]. In our research, we used *E. coli* to express Smad2/3/4 recombinant protein. However, *E. coli* system could not express recombinant protein with phosphorylation. Therefore, we could not get the detailed information of the phosphorylation effect on Smad2/3/4 LLPS. We are trying to use the HEK293T cell to express Smad2/3/4 recombinant proteins with phosphorylation modification. This may give us more information about the Smad2/3/4 LLPS. Smad family members not only include Smad2, Smad3 and Smad4, but also contain Smad1, Smad5, Smad7, Smad8 and Smad6 [47]. We are also interested in whether these proteins can undergo LLPS.

Previous studies showed that transcription factor can undergo LLPS with mediator to activate gene expression [15, 16]. Our research found that Smad2/3/4 complex could undergo phase separation, so we believed that Smad2/3/4 complex underwent phase separation in transcription activity. We performed a series of experiments to find that Smad2/3/4 complex could regulate TAT expression then inhibit HCC progress. It is reported that Smad3 and Smad4 can bind directly to DNA(Smad2 can’t), and the affinity between Smad3/4 and DNA is relatively weak and need another transcription factor to regulate gene expression[48, 49]. We are also interested in whether other proteins take part in Smad2/3/4 complex undergoing LLPS. We conducted Smad3 and Smad4 CoIP experiment and mass spectrometry analysis (data not shown), hoped to get more information about this.

Studies have shown that activation of TAT in the tyrosine metabolic pathway influences the treatment resistance of glioblastoma core [50]. The *TAT* gene encodes the mitochondrial protein tyrosine aminotransferase present in liver, which breaks down tyrosine into p-hydroxyphenylpyruvate. Although aberrant expression of TAT in HCC has been shown to be associated with a poor prognosis [31], currently, there is little evidence on how tyrosine metabolism affects cancer progress. To find the cause of the low expression of TAT in HCC, we focused on the TGF-β/Smad2/3/4 signaling pathway, which also acted as a tumor suppressor in cancer [51, 52]. Our data connected TGF-β signaling pathway and TAT in HCC progress, which brought new insights in TGF-β signaling pathway function in HCC progress.

Above all, we found that Smad2/3/4 could undergo liquid–liquid phase separation to active *TAT* expression. We experimentally verified a significant link between TAT and Smad2/3/4 complex in HCC progress. In addition, we found that Smad2/3/4 complex could active caspase-9 to induce hepatoma cell apoptosis through regulating TAT. This result contributes to deeper understanding of the role of TGF-β signaling pathway in HCC progress. Meanwhile, it also brings new insights into the mechanism of hepatocellular carcinoma.

## Conclusions

Our results showed that TAT showed low expression in clinical HCC samples. Notably, TAT was found to be a novel target gene of Smad2/3/4 complex, and Smad2/3/4 could undergo phase separation to active TAT gene expression. Smad2/3/4 activated caspase-9 through regulating TAT to induce hepatoma cell apoptosis. Our research suggested that inhibiting Smad2/3/4 LLPS might become a new strategy to inhibit HCC progress. Above all, we found that Smad2/3/4 complex could undergo liquid liquid phase separation to active TAT gene expression, and active caspase-9 to induce hepatoma cell apoptosis through regulating TAT to inhibit HCC progress.

### Supplementary Information

Supplementary material 1: Figure S1. Regulated Smad2, Smad3 and Smad4 expression level in HCC cell lines. (A) ShRNA knockdown efficiency of Smad2 was examined by qPCR and western blot analysis in HUH7 cells. (B) qPCR and western blot analysis showed the overexpressed of Smad2 by eukaryotic expression plasmid in HUH7 cells. (C-D) The expression level of Smad2 was examined by qPCR and western blot analysis in HepG2 cells. (E-F) The expression level of Smad3 was examined by qPCR and western blot analysis in HUH7 cells. (G-H) The expression level of Smad3 was examined by qPCR and western blot analysis in HepG2 cells. (I-J) The expression level of Smad4 was examined by qPCR and western blot analysis in HUH7 cells. (K-L) The expression level of Smad4 was examined by qPCR and western blot analysis in HepG2 cells. 3μg and 5μg is represented the quality of overexpression vector in each hole of 6-Wells plats. *P*<0.05, *; *P*<0.01,**; *P*<0.001,***; *P*<0.0001, ****. TGF-β1 (150 pM) was added in medium to activate Smads.

Supplementary material 2: Figure S2. Smad2/3/4 complex could inhibit HCC cell proliferation through TAT in HepG2 cells. (A-C) CCK analysis showed that knock down Smad2 (A), Smad3 (B) and Smad4 (C) could promote HepG2 cells proliferation. (D) Colony formation analysis showed that knock down Smad2, Smad3 and Smad4 could promote HepG2 cells proliferation. (E-G) CCK analysis showed that overexpressing TAT could inhibit HepG2 cells proliferation and knock down Smad2 (E), Smad3 (F) and Smad4 (G) could induce a compensatory effect. (H-J) Colony formation analysis showed that overexpressing TAT could inhibit HepG2 cells proliferation and knock down Smad2 (H), Smad3 (I) and Smad4 (J) could induce a compensatory effect. 5μg is represented the quality of overexpression vector in each hole of 6-Wells plats. *P*<0.05, *; *P*<0.01,**; *P*<0.001,***; *P*<0.0001, ****. TGF-β1 (150 pM) was added in medium to activate Smads.

Supplementary material 3: Figure S3. Smad2/3/4 could active caspase-9 through TAT in HepG2 cells. (A-C) Flow cytometry analysis showed that upregulated Smad2 (A), Smad3 (B) and Smad4 (C) could induce HepG2 cell apoptosis. (D-F) Upregulated Smad2 (D), Smad3 (E) and Smad4 (F) could active caspase-9 in HepG2 cells. (G-I) Flow cytometry analysis showed that upregulated TAT could induce apoptosis, but knocking down Smad2 (G), Smad3 (H), Smad4 (I) could inhibit apoptosis in HepG2 cells. (J-L) Upregulated TAT could active caspase-9, but knocking down Smad2 (J), Smad3 (K), Smad4 (L) could inhibit the caspase-9 active in HepG2 cells. 3μg and 5μg is represented the quality of overexpression vector in each hole of 6-Wells plats. TGF-β1 (150 pM) was added in medium to activate Smads.

## Data Availability

There no new data were created.
