# Supplementary figures and images for: Smad2/3/4 complex could undergo liquid liquid phase separation and induce apoptosis through TAT in hepatocellular carcinoma
Source: Cancer Cell Int. 2024 May 21;24:176. doi: 10.1186/s12935-024-03353-x (PMC11106862; doi:10.1186/s12935-024-03353-x)

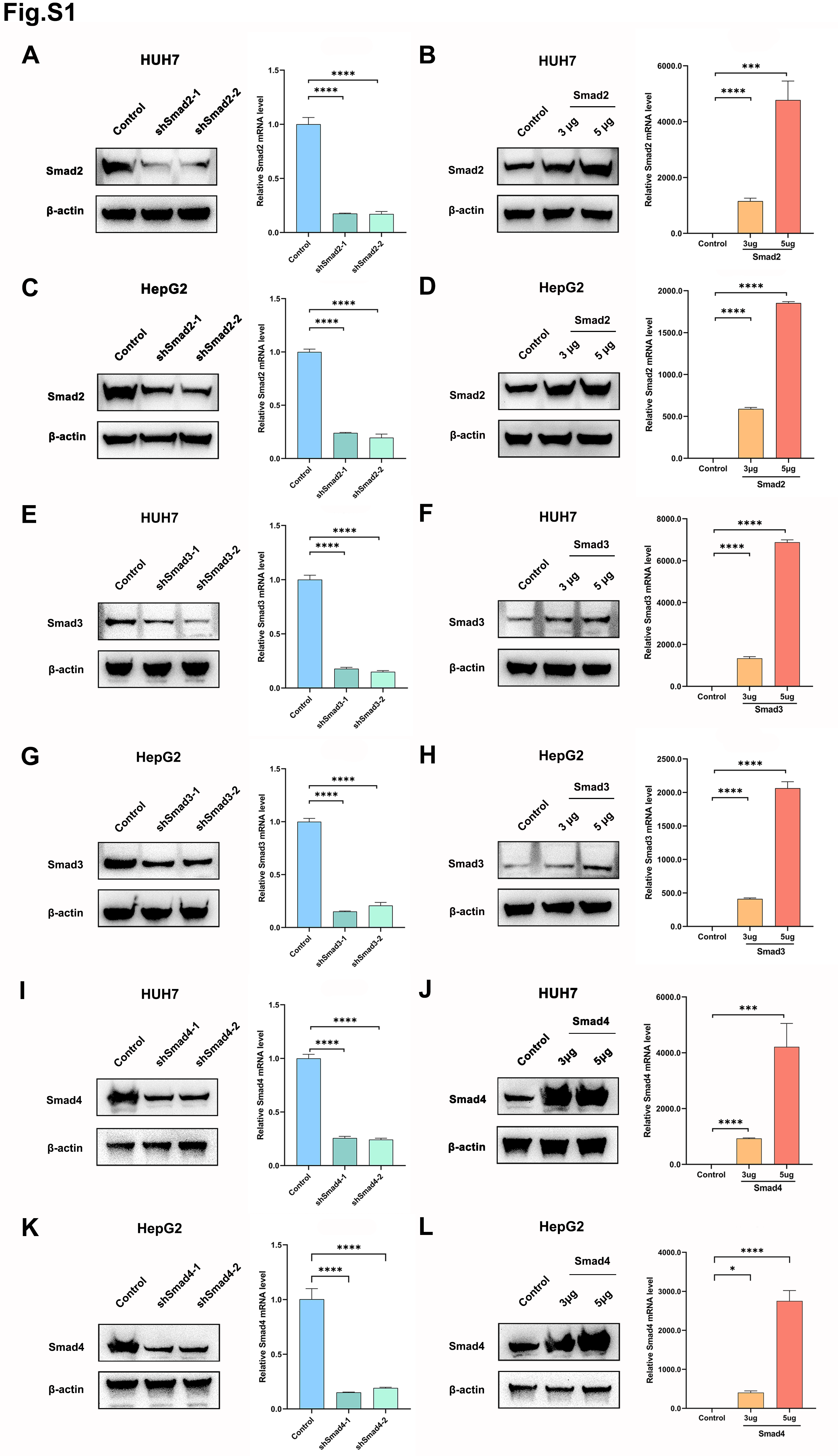

Supplement: Supplementary file 1 — Supplementary material 1: Figure S1. Regulated Smad2, Smad3 and Smad4 expression level in HCC cell lines. (A) ShRNA knockdown efficiency of Smad2 was examined by qPCR and western blot analysis in HUH7 cells. (B) qPCR and western blot analysis showed the overexpressed of Smad2 by eukaryotic expression plasmid in HUH7 cells. (C-D) The expression level of Smad2 was examined by qPCR and western blot analysis in HepG2 cells. (E-F) The expression level of Smad3 was examined by qPCR and western blot analysis in HUH7 cells. (G-H) The expression level of Smad3 was examined by qPCR and western blot analysis in HepG2 cells. (I-J) The expression level of Smad4 was examined by qPCR and western blot analysis in HUH7 cells. (K-L) The expression level of Smad4 was examined by qPCR and western blot analysis in HepG2 cells. 3μg and 5μg is represented the quality of overexpression vector in each hole of 6-Wells plats. P<0.05, *; P<0.01,**; P<0.001,***; P<0.0001, ****. TGF-β1 (150 pM) was added in medium to activate Smads. [file 12935_2024_3353_MOESM1_ESM.tif]

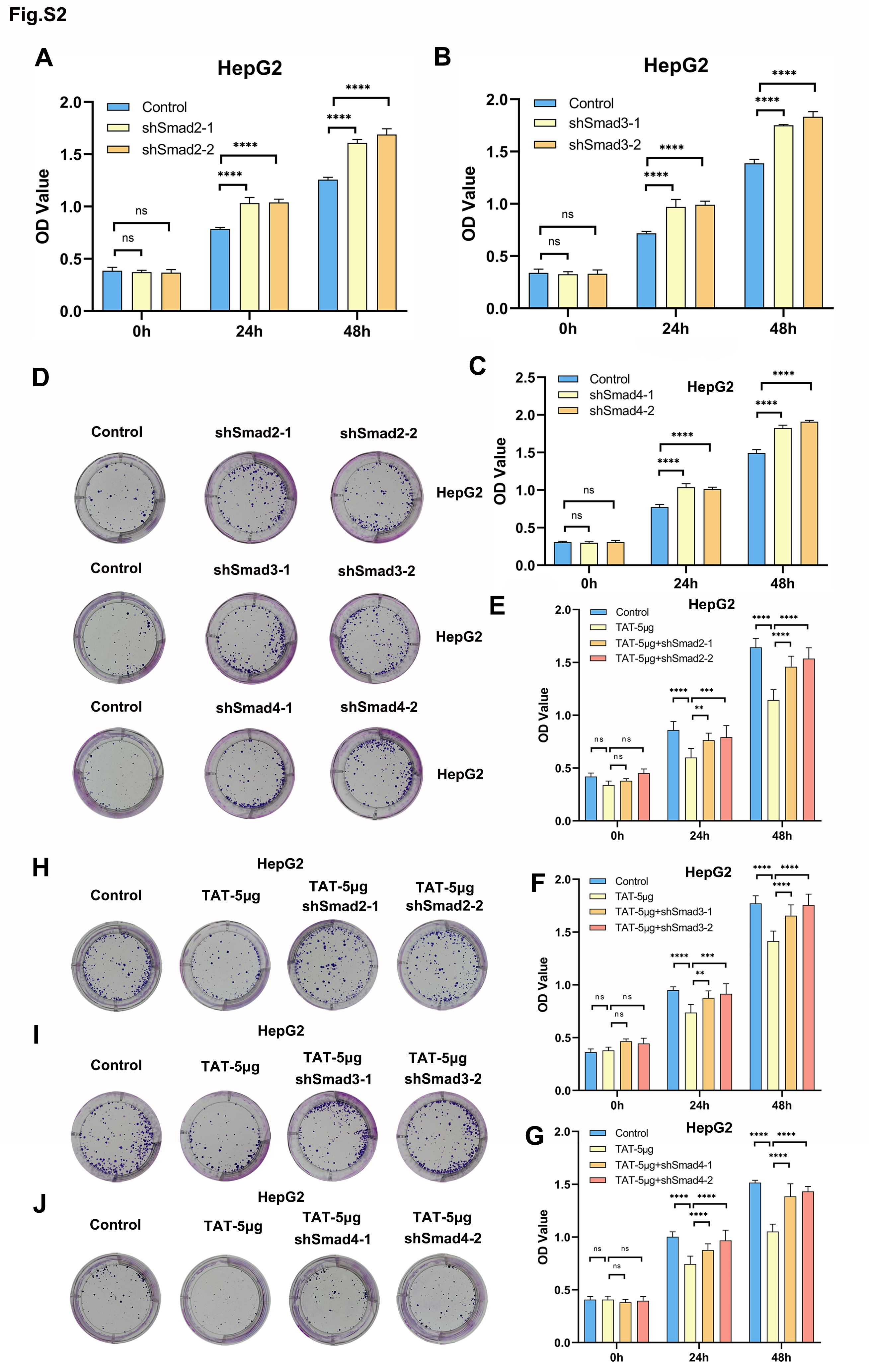

Supplement: Supplementary file 2 — Supplementary material 2: Figure S2. Smad2/3/4 complex could inhibit HCC cell proliferation through TAT in HepG2 cells. (A-C) CCK analysis showed that knock down Smad2 (A), Smad3 (B) and Smad4 (C) could promote HepG2 cells proliferation. (D) Colony formation analysis showed that knock down Smad2, Smad3 and Smad4 could promote HepG2 cells proliferation. (E-G) CCK analysis showed that overexpressing TAT could inhibit HepG2 cells proliferation and knock down Smad2 (E), Smad3 (F) and Smad4 (G) could induce a compensatory effect. (H-J) Colony formation analysis showed that overexpressing TAT could inhibit HepG2 cells proliferation and knock down Smad2 (H), Smad3 (I) and Smad4 (J) could induce a compensatory effect. 5μg is represented the quality of overexpression vector in each hole of 6-Wells plats. P<0.05, *; P<0.01,**; P<0.001,***; P<0.0001, ****. TGF-β1 (150 pM) was added in medium to activate Smads. [file 12935_2024_3353_MOESM2_ESM.tif]

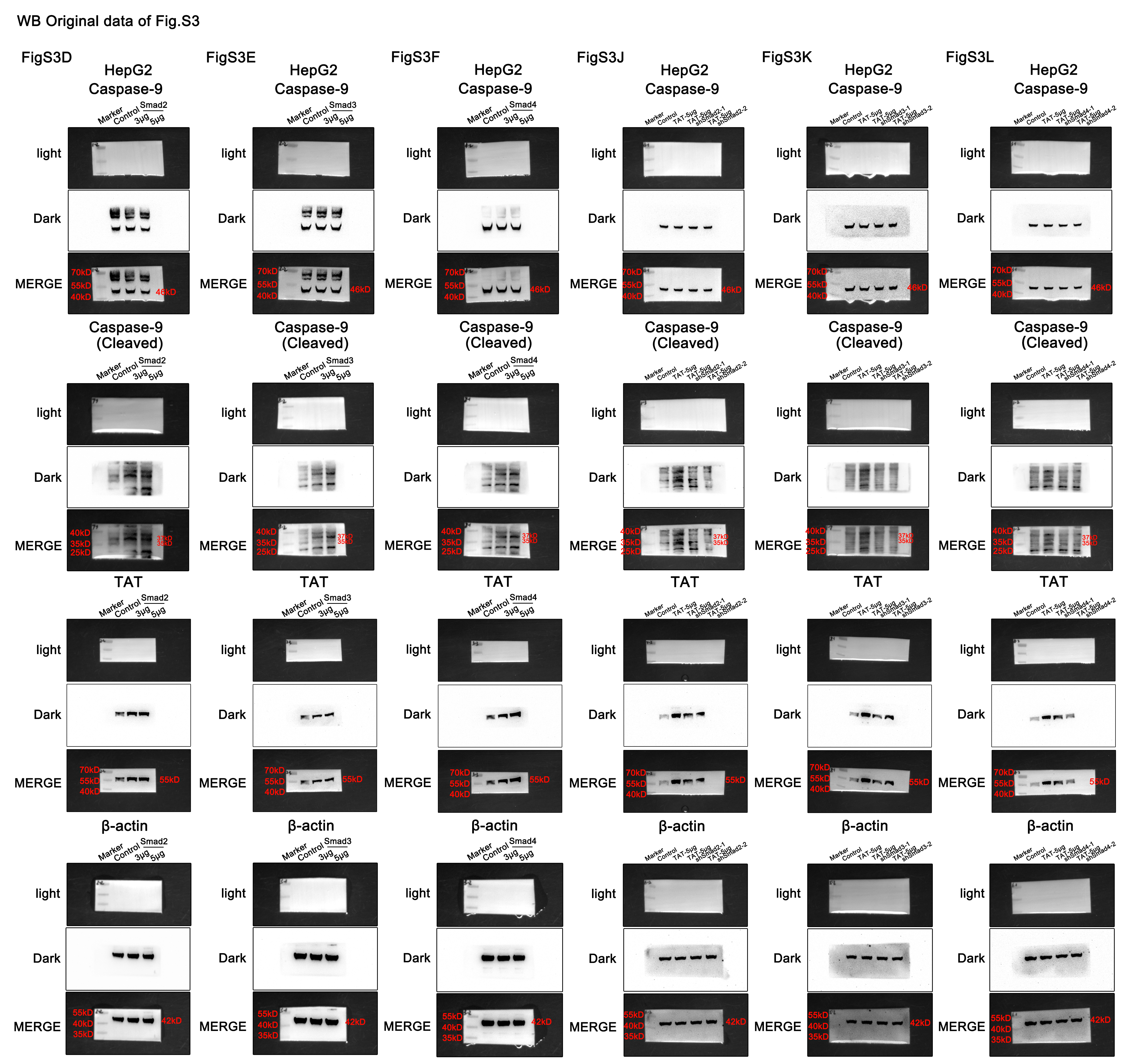

Supplement: Supplementary file 3 — Supplementary material 3: Figure S3. Smad2/3/4 could active caspase-9 through TAT in HepG2 cells. (A-C) Flow cytometry analysis showed that upregulated Smad2 (A), Smad3 (B) and Smad4 (C) could induce HepG2 cell apoptosis. (D-F) Upregulated Smad2 (D), Smad3 (E) and Smad4 (F) could active caspase-9 in HepG2 cells. (G-I) Flow cytometry analysis showed that upregulated TAT could induce apoptosis, but knocking down Smad2 (G), Smad3 (H), Smad4 (I) could inhibit apoptosis in HepG2 cells. (J-L) Upregulated TAT could active caspase-9, but knocking down Smad2 (J), Smad3 (K), Smad4 (L) could inhibit the caspase-9 active in HepG2 cells. 3μg and 5μg is represented the quality of overexpression vector in each hole of 6-Wells plats. TGF-β1 (150 pM) was added in medium to activate Smads. [file 12935_2024_3353_MOESM3_ESM.tif]
